# Comparative Study of the Structural Properties, Color, Bioactive Compounds Content and Antioxidant Capacity of Aerated Gelatin Gels Enriched with Cryoconcentrated Blueberry Juice during Storage

**DOI:** 10.3390/polym12122769

**Published:** 2020-11-24

**Authors:** Nidia Casas-Forero, Patricio Orellana-Palma, Guillermo Petzold

**Affiliations:** 1 Laboratory of Cryoconcentration, Department of Food Engineering, Universidad del Bío-Bío, Av. Andrés Bello 720, Casilla 447, Chillán 3780000, Chile; nidiacf@gmail.com; 2 Doctorado en Ingeniería de Alimentos, Universidad del Bío-Bío, Av. Andrés Bello 720, Casilla 447, Chillán 3780000, Chile; 3 Department of Biotechnology, Universidad Tecnológica Metropolitana, Las Palmeras 3360, Ñuñoa, Santiago 7800003, Chile

**Keywords:** non-thermal juice concentration, fruit juice concentrate, gel, aeration, mechanical properties, preservation, phenolic content, antioxidant capacity

## Abstract

Cryoconcentrated blueberry juice (CBJ) was incorporated into aerated gelatin gel and the effects on the mechanical properties, phenolic compounds and antioxidant activity (AA) were evaluated at day 1 and day 28 under refrigerated storage. At day 1, 8 g of gelatin gel and 40 g of CBJ (called M5) exhibited a soft texture and heterogeneous and non-spherical small bubbles, with values close to 10.5, 8.0 and 7.1 N, for hardness, gumminess and chewiness, respectively. M5 presented an increase of approximately 1.7, 1.9 and 1.9, and 1.2, 1.8, 2.1 and 1.3 times in comparison to the other samples, for total polyphenol, anthocyanin and flavonoid contents, and individual phenolic compounds, 2,2-diphenyl-1-picrylhydrazyl (DPPH), ferric reducing antioxidant power (FRAP) and oxygen radical absorbance capacity (ORAC) assays, respectively. At day 28, the samples showed a weakening of the 3D network, with high degradation of phenolic compounds and AA due to the oxidation, polymerization and syneresis. Therefore, CBJ might be an interesting functional ingredient to add to (aerated and non-aerated) gelatin gel without affecting its properties, and thus different food products with high nutritional values and without added artificial sweeteners could be developed. Additionally, the gelatin gel/CBJ combinations might be suitable for additive manufacturing as a coating of food matrices.

## 1. Introduction

Gelatin is a versatile biomaterial derived from collagen that allows the fabrication of a porous and 3D solid matrix (gel) with excellent mechanical properties, high absorption capacity, high durability and stability, low price and high biocompatibility and biodegradability [1,2,3]. Thereby, the gels (or hydrogels) are a 3D cross-linked polymer network that can absorb and/or retain water in the interstitial spaces between the polymer chains. Thus, these matrices have been used in different engineering applications [4]. Specifically, the formation of gelatin gels depends on the temperature, since a temperature below 35 °C means that the disordered peptide chains will be ordered, and thus a collagen triple-helix structure can be formed, which acts as a cross-linked gel matrix stabilized by intermolecular hydrogen bonds [5].

Thus, aerogel products are attractive porous solid materials, and these can be manufactured by replacing the liquid with gas into a solid gel matrix (as gelatin gels) through various methods such as membranes, ultrasound, chemical reactions or mechanical agitation (conventional process), resulting in a gel with uniform pore sizes, low density, high porosity and high specific surface area [6]. 

Specifically, in food products, the aeration can be dispersed in liquid or viscous solutions as air bubbles, and thus, depending on the bubble distribution inside the food, it is possible to obtain a food product with textures from soft and rubbery to hard and brittle [7]. Consequently, aerated gelatin gels have gained considerable attention among children and adults due to the textural properties and sensory characteristics, and in addition, the aerogel products promote satiety and low caloric density [8]. Furthermore, the aerated configuration can facilitate the creation of new structures, allowing for these products to be used as an encapsulating matrix or as a protective coating on various components such as vitamins, minerals, flavors, bioactive peptides and phytochemicals as phenolic and antioxidant compounds [9,10].

In recent years, cryoconcentration (CC) has gained considerable attention since it is an emerging and interesting technology that uses low temperatures to increase soluble solids [11] and to retain heat-sensitive compounds of diverse food solutions [12]. Specifically, the CC process is based on freezing the water until reaching the eutectic point of the solution. Hence, once the crystals are formed, the unfrozen solution, i.e., the cryoconcentrated fraction, can be separated from the frozen fraction (ice crystals) [13]. Hence, CC has been characterized by high concentrations at low temperatures that help to reduce the loss of physicochemical properties, bioactive and volatile compounds and antioxidant activity in comparison to other concentration technologies [14]. Likewise, CC has shown a considerable increase in antioxidant and cytotoxic (cancerous cells and non-tumorigenic cells) activity in a cryoconcentrated sample (potato juice), indicating that CC presents advantages at the cellular level compared to other technologies [15]. Thereby, these favorable effects have been explored in fruit juices such as orange juice [16,17,18], strawberry juice [19], grape juice [20], blueberry juice [21,22,23], apple juice [24,25,26] and pineapple juice [27]. Moreover, numerous studies have reported the application of CC in other food products such as feijoa pulp [28], grape must [29], bifidobacteria entrapped in cryoconcentrated solution [30] and corn starch incorporated in cryoconcentrated yerba mate [31], highlighting the versatility and potential of CC as an alternative non-thermal concentration technology.

However, the application of cryoconcentrated samples to enrich food matrices has been poorly investigated. To date, cryoconcentrated juice has only been informed to enrich dried apple slices [32] and natural yogurt [33]. These studies indicate a significant improvement in quality attributes such as physicochemical properties, bioactive compounds and antioxidant activity, since the cryoconcentrate presented high bioactive components content and attractive color, and thus it improved various solid matrix characteristics. Therefore, to fill this knowledge gap, studies about the incorporation of cryoconcentrated juice inside different food matrices and, particularly, the incorporation in gelatin-based confectionery products are required, and this would allow the development of novel food products.

Therefore, the aim of this study was to investigate the effects of cryoconcentrated blueberry juice (CBJ) incorporation inside aerated gelatin gels in terms of structural properties, color, bioactive compounds content and antioxidant activity, which could provide with an insight into the development of new aerated gel-based products enriched with cryoconcentrated juice. Hence, the innovation and novelty of this study are based on the use of cryoconcentrated juice as a functional ingredient in an aerated food matrix (aerated gelatin gels). As an important point, there is no prior study that has evaluated the addition of cryoconcentrated juice to an aerated food.

## 2. Materials and Methods 

### 2.1. Materials 

Fresh blueberries (*Vaccinium corymbosum* L., cv. *Brigitta*) were purchased from a supermarket in Chillán city (Región del Ñuble, Chile) and the fruits were stored under refrigeration (≈4 °C) until processing. Commercial bovine skin gelatin type-B (catalogue number: G9382, protein content > 70%, ∼225 g Bloom and molecular weight: 50 kDa), Folin–Ciocalteu reagent, sodium carbonate (Na_2_CO_3_), potassium chloride (KCl), sodium acetate (CH_3_COONa), sodium nitrite (NaNO_2_), aluminum chloride (AlCl_3_), sodium hydroxide (NaOH), acid gallic, cyanidin-3-glucoside, (+)-catechin, 1,1-Diphenyl-2-picryl-hydrazyl (DPPH), 2,4,6-tris(2-pyridyl)-S-triazine (TPTZ), fluorescein solution, 2,2′-azobis (2-methylpropionamidine) dihydrochloride (AAPH), (±)-6-hydroxyl-2,5,7,8-tetramethyl-2-carboxylic acid (Trolox) and each individual phenolic compound standard were purchased from Sigma-Aldrich (Sigma-Aldrich Inc., St. Loius, MO, USA). Distilled water was used throughout. All reagents used were of analytical grade. 

### 2.2. CBJ Preparation

The blueberry juice was extracted using a tabletop juicer, and the juice was immediately filtered through a nylon cloth (0.8 mm mesh) to remove solid material (skin and seeds). The concentration was carried out by centrifugal block CC (CBCC) at three cycles as described previously by Casas-Forero et al. [34], with some modifications. Briefly, the juice (45 mL) was placed into plastic centrifugal tubes isolated with foamed polystyrene (8 mm thickness, thermal conductivity K = 0.035 W/mK) to produce an axial freezing, and later, the tubes were frozen in a static freezer (280, M&S Consul, Sao Paulo, Brazil) overnight at −20 °C. Once the freezing process was completed, the CBJ was separated from the ice fraction by centrifugation (Eppendorf 5430R, Hamburg, Germany) at 15 °C for 20 min at 4600 rpm. Thus, this procedure is equivalent to the first cycle. Specifically, three CC cycles were realized, i.e., the CBJ sample from the first cycle was used as feed solution to a second and third cycle with the procedure described above (freezing and separation by centrifugation). In the third cycle, the total soluble solid content (TSSC) in CBJ reached a value close to 45 °Brix.

### 2.3. CBJ Incorporation into Aerated Gelatin Gels

Preliminary experiments (data not shown) were performed to determine an optimal combination between the CBJ and gelation gel solution to avoid damaging the physical integrity of the aerated CBJ–gelatin gel sample. Thus, a solid and compact structure was reached at a final TSSC concentration of approximately 19 °Brix. Therefore, different CBJ and gelatin gel proportions were mixed (with a final TSSC value of 19 °Brix), as shown in Table 1.

The gelatin gel solution was prepared by the method described by Dai et al. [35], with modifications. Gelatin powder was hydrated in distilled water (3 g/100 mL) for 10 min, the solution was maintained with constant agitation using a magnetic stirrer at 60 °C for 5 min and then, later, the temperature was decreased to 40 °C; immediately after, the CBJ was added to the gel solution, and it was mixed for 5 min to achieve the complete incorporation of CBJ in the gel solution. Immediately, air bubbles were incorporated in the CBJ–gel solution by agitation for 9 min using an Oster^®^ mixer (Oster 2532, 250-watt, 6-Speed, Rianxo, Spain). Finally, the aerated CBJ–gel solution was placed in cylindrical plastic containers (diameter = 34 mm and height = 15 mm), and the samples were refrigerated at 4 °C (overnight) to form a solid gel. Quality analyses were performed on day 1 (control) and day 28 under storage at 4 °C in a refrigerated incubator (FOC 215E, Velp Scientific Inc., Milano, Italy).

### 2.4. Apparent Viscosity

The apparent viscosity (kPa∙s) of the aerated CBJ–gelatin gel samples was determined at 30 °C in a rotational-type rheometer (Physica MCR300, Anton Paar GmbH, Stuttgard, Germany) equipped with a parallel plate geometry (diameter 50 mm) and a gap of 0.8 mm between the two plates, varying the shear rate from 1 to 300 s^−1^ [36]. Four repetitions were performed for each sample.

### 2.5. Density and Gas Hold-Up Measurements

The density of the aerated CBJ–gelatin gel (*ρ_acg_*) was measured by the flotation method [7]. A corked glass flask was weighed with/without distilled water at 25 °C, then the sample was placed in a glass flask with distilled water at 25 °C, corked and the weight was measured and recorded. The density of the sample was calculated according to Equation (1).

(1) $$\rho_{acg}\left(\frac{\mathrm{kg}}{m^{3}}\right)=\frac{\rho_{w}\times m_{acg}}{m_{acg}+m_{g}-m_{g+acg}}$$

where *ρ_w_* is the density of water at 25 °C (997.05 kg/m^3^), *m_acg_* is the mass of aerated CBJ–gelatin gel (kg), m_g_ is the mass of the glass flask with water at 25 °C (kg) and *m_g+acg_* is the mass of the glass flask with water and aerated CBJ–gelatin gel (kg). The density of CBJ–gelatin gel (*ρ_cg_*) was obtained with the same procedure as aerated CBJ–gelatin gel (*ρ_acg_*).

Gas hold-up (*ε*) was obtained by comparing the density of aerated CBJ–gelatin gel (*ρ_acg_*) and CBJ–gelatin gel (*ρ_cg_*). The *ε* was calculated according to Equation (2).

(2) $$\varepsilon \left(\%\right)=\left(1-\frac{\rho_{acg}}{\rho_{cg}}\right)\times 100$$

where *ρ_acg_* is the density of aerated CBJ–gelatin gel and *ρ_ag_* is the density of CBJ–gelatin gel.

### 2.6. Texture Profile Analysis (TPA)

The TPA of the samples was determined according to Cai et al. [37], with modifications. A texture analyzer (TA.XT plus100, Stable Micro Systems Ltd., Surrey, UK) coupled to a PC with Exponent Connect software (version 7.0.3.0, Hamilton, MA, USA) was used for the tests. A double compression was carried out with a 50 mm diameter aluminum cylindrical probe (P50), which was pressed into the samples by a 5.0 kg load cell at a crosshead speed of 1.0 mm/s and an interval between compressions of 3.0 s. Each sample was compressed to 50% of its original height. Textural parameters such as hardness, cohesiveness, gumminess, chewiness and springiness were generated from the force–time graphic.

### 2.7. Bubble Size Quantification by Image Analysis

The bubble size distribution of aerated CBJ–gelatin gel was quantified by the method reported by Orrego et al. [9]. Each aerated CBJ–gelatin gel sample was cut into slices (thickness of 3 mm) and ten images for each sample were acquired using an Olympus Trinocular Microscope (Olympus Co., Tokyo, Japan) coupled to a digital camera, Olympus LC 20 (Olympus Co., Munster, Germany). The images were acquired at 1600 × 1200 pixels. The boundaries of bubbles were manually traced, and the area of each bubble was measured using ImageJ software (Version 1.52i, National Institute of Health, Bethesda, MD, USA). 

### 2.8. Color Analysis

The color properties were determined in a portable chromameter (CR–400, Konica Minolta, Osaka, Japan). The CIELab coordinates (L*: darkness–lightness, a*: green–red axis, b*: blue–yellow axis, C_ab_*: chroma and h_ab_*: hue) were determined using the standard illuminant D65 and an observer angle of 10°. Moreover, the total color difference (ΔE*), i.e., the Euclidean distance between two points in a 3D space, was calculated to find any difference between day 1 and day 28. The ΔE* was calculated according to Equation (3) [38].

(3) $$\Delta E^{*}=\sqrt{{\left(\Delta L^{*}\right)}^{2}+{\left(\Delta a^{*}\right)}^{2}+{\left(\Delta b^{*}\right)}^{2}\;\;\;}$$

where ΔL* = (L* − *L*_0_*), Δa* = (a* − *a*_0_*) and Δb* = (b* − *b*_0_*). Subscript 0 indicates color of day 1.

### 2.9. Determination of Total Bioactive Compounds Content (TBCC)

Total polyphenol content (TPC) was measured according to the Folin–Ciocalteau method described by Waterhouse [39], with minor modifications. Gallic acid (GA) was used as standard. An amount of 100 μL of the sample was mixed with 500 μL of 10-fold diluted Folin–Ciocalteu reagent. Then, the solution was vigorously mixed with 1500 μL of Na_2_CO_3_ (20% *w*/*v*). After 90 min in the dark at room temperature (incubation), the absorbance was recorded at 760 nm. TPC was calculated as milligrams of GA equivalents (GAE) per 100 g of dry matter (mg GAE/100 g d.m.).

Total anthocyanin content (TAC) was quantified by the differential pH method according to Lee et al. [40], with some modifications. Cyanidin-3-O-glucoside (C3G) was used as standard. An amount of 200 μL of sample was mixed with 800 μL of KCl (pH 1.0, 0.025 M) and 800 μL of CH_3_COONa (pH 4.5, 0.4 M) buffers. After 30 min in the dark at room temperature (incubation), the absorbance was measured at 510 and 700 nm. TAC was calculated as milligrams of C3G equivalents per 100 g of dry matter (mg C3G/100 g d.m.).

Total flavonoid content (TFC) was determined using the colorimetric method described previously by Dewanto et al. [41], with slight modifications. Catechin (C) was used as standard. An amount of 250 μL of sample was mixed with 1250 μL of distilled water and 75 μL of NaNO_2_ (5% *w*/*v*). After 6 min, the solution was combined with 150 μL of AlCl_3_ (10% *w*/*v*), 500 μL of NaOH (1.0 M) and 275 μL of distilled water. After 10 min in the dark at room temperature (incubation), the absorbance was measured at 510 nm. TFC was calculated as milligrams of C equivalents (CEQ) per 100 g of dry matter (mg CEQ/100 g d.m.).

TPC, TFC and TAC were evaluated using a UV–Vis spectrophotometer (T70, Oasis Scientific Inc., Greenville, SC, USA).

### 2.10. Determination of Antioxidant Activity (AA)

The DPPH assay was assessed using the method reported by Brand-Williams et al. [42], with minor modifications. An amount of 150 µL of sample was mixed with 2850 µL of DPPH methanolic solution. The mixture was kept in the dark at room temperature for 30 min (incubation), and the absorbance was measured at 515 nm.

The ferric reducing antioxidant power (FRAP) assay was performed according to Benzie and Strain [43], with some modifications. Briefly, FRAP reagent was prepared with 50 mL of CH_3_COONa buffer (pH 3.6, 300 mM), 5.0 mL of TPTZ (10 mM in hydrochloric acid (40 mM)) and 5.0 mL of FeCl_3_·6H_2_O (20 mM) (10:1:1 ratio), and then the solution was incubated at 37 °C. An amount of 150 µL of sample was mixed with 2850 µL of FRAP reagent. The solution was kept in the dark at 37 °C for 30 min (incubation), and the absorbance was measured at 593 nm.

DPPH and FRAP assays were quantified on a UV–Vis spectrophotometer (T70, Oasis Scientific Inc., Greenville, SC, USA).

The oxygen radical absorbance capacity (ORAC) method was determined using the method reported by Prior et al. [44]. Specifically, 45 μL of sample, 175 μL of fluorescein solution (167 nM) and 50 μL of AAPH (97.2 mM) were placed into black 96-well microplates and incubated at 37 °C for 30 min. The fluorescence intensity was measured every 3 min for 300 min at an excitation wavelength of 485 nm and with emission set to 535 nm using a multimode plate reader (Victor TM X2, Perkin Elmer, Hamburg, Germany).

For all assays, Trolox (T) was used as the standard curve, and the results were expressed as µmol Trolox equivalents (TE) per 100 g of dry matter (µM TE/100 g d.m.). 

### 2.11. Analysis of Individual Phenolic Compounds (IPC) by High-Performance Liquid Chromatography (HPLC)

HPLC analysis was conducted in accordance with the previously proposed method by Ruiz et al. [45]. An amount of 50 μL of sample was injected into an HPLC instrument (Series 200, Perkin Elmer, Boston, MA, USA), and the analyses were carried out on a Purospher STAR^®^ 100 RP-18e column (125 × 4.0 mm, 5.0 μm particle size). The mobile phases were composed of water/acetonitrile/aqueous formic acid (87:3:10% *v*/*v*/*v*) (phase A) and water/acetonitrile/aqueous formic acid (87:3:10% *v*/*v*/*v*) (phase B), at a flow rate of 0.8 mL/min, and the absorbance was measured simultaneously at 280 and 520 nm. The IPC in aerated CBJ–gelatin gels were identified using the UV–Vis absorption spectra and retention times of the standard compounds. The IPC were quantified as delphinidin, cyanidin, malvidin, epigallocatechin gallate, epicatechin, quercetin, myricetin and caffeic acid. The results were expressed as µmol of standard per 100 g of dry matter (µM/100 g d.m.).

### 2.12. Determination of Recovery Percentage (RP)

The *RP* of bioactive compounds (TPC, TAC, TFC and IPC) in the aerated CBJ–gelatin gels at day 0 and day 28 was calculated according to Pineda-Vadillo et al. [46] using Equation (4).

(4) $$RP\left(\%\right)=\left(\frac{Q_{acg}}{Q_{cbj}}\right)\times 100$$

where *Q_acg_* is the bioactive compound content in aerated CBJ–gelatin gel and *Q_cbj_* is the bioactive compound content in CBJ added to the gelatin gel solution.

### 2.13. Statistical Analysis

All the experiments were carried out in triplicate at 25 °C, and the results were reported as the mean values ± standard deviation (SD). Statistical analyses were performed with Statgraphics Centurion XVI^®^ software (StatPoint Technologies Inc., Warrenton, VA, USA). One-way ANOVA, the least significant difference (LSD) test and the Student’s *t*-test were used for significant differences between means (*p* ≤ 0.05).

## 3. Results and Discussion

### 3.1. Apparent Viscosity, Density and Gas Hold-Up Measurements

Table 2 summarizes the apparent viscosity, density and gas hold-up measurements obtained with different gelatin gel/CBJ combinations. 

For apparent viscosity, from M1 to M5, a progressive decrease in the samples was acquired as the amount of gelatin gel solution decreased and the amount of CBJ increased, demonstrating that the gelatin gel/CBJ combination had a direct and an opposite relationship on the apparent viscosity, respectively. Thus, M1 (14 g gelatin gel/20 g CBJ) obtained the highest apparent viscosity, with a value close to 31 (kPa·s), indicating that the amount of gelatin gel had an important influence on the final characteristic in each sample, since M5 (8 g gelatin gel/40 g CBJ) had a value of approximately 7.1 (kPa·s), which is equivalent to ≈23% of M1. Specifically, this performance could be connected to the gelatin gel/CBJ mixture, since a significant decrease in the amount of gelatin gel solution generates a weakening of the final configuration due to the low amount of molecules that interact to form triple-helical structures. Moreover, an increase in the CBJ solution hinders the gelling process, since it intervenes in the molecules’ interaction, and thus the CBJ obstructs the formation of the gelatin network [47]. Therefore, a high amount of CBJ has an antagonistic effect on the gelling process with a low amount of gelatin gel solution, leading to a fragile structure, which is prone to severe internal changes when subjected to external factors. Even so, M5 showed a viscosity in the range of commercial gelatin gels (2–7 (kPa·s)) [48], allowing a marketable advantage to develop aerated gelatin gel-based products with a liquid ingredient such as cryoconcentrated juice.

In terms of density, a similar behavior to viscosity was also observed since the values were decreased significantly from M1 to M5. Hence, M1 produced a higher density value than the other samples, with ≈511 (kg/m^3^), while M5 presented a value close to 349 (kg/m^3^), and this indicates a decrease of 32% with respect to M1. Hence, these downward trends could be explicated by the gradual decrease in the amount of gelatin gel solution, since the typical 3D organization depends on the weight of the gelatin gel solution, representing a direct proportionality between mass and density in the sample elaboration [49]. Besides, the amount of CBJ (20–40 g) added to the gel solution did not have a significant effect on the density of the final product, and this can be elucidated by the slight increase in the density of the CBJ sample (third cycle), since it was 9% lower than initial fresh blueberry juice (data not shown), i.e., the addition of CBJ does not compensate the weight loss by decreasing the amount of gelatin gel solution. Moreover, the density values in the aerated CBJ–gelatin gels were lower than CBJ–gelatin gel samples, since the aerated samples presented less than 50% of the density values of CBJ–gelatin gels, relating to the incorporation of air bubbles, since the air allows reducing the weight of the gelatin gel sample [7]. Thus, the viscosity and density values specify that the amounts of gelatin gel and CBJ used are excellent to elaborate an aerated gelatin gel enriched with CBJ, since the samples presented a final structure similar (visual aspect) to commercial products.

On the other hand, the ε values showed a contrary effect, since the values had an increase as the amount of gelatin gel solution decreased and the amount of CBJ increased, with values of approximately 49%, 52%, 57%, 64% and 67%, for M1, M2, M3, M4 and M5, respectively. Specifically, this upward tendency could be correlated with the continuous phase of the gelatin gel solution, since the gelatin gel creates an energy barrier, and it depends on the initial amount of gel solution. Hence, a high initial amount makes the incorporation of air bubbles inside the gel solution during the agitation process difficult [50]. Thereby, the energy barrier decreased with the decrease in the amount of gel gelatin solution, allowing a high aggregation of air bubbles into the sample, reflecting in the high ε value for M5. 

The values are in accordance with Jakubczyk et al. [7], Orrego et al. [9] and O’Chiu and Vardhanabhuti [51], who added air bubbles in different gelatin gel samples such as agar–fructose gels, whey protein gels and dairy gels, respectively, indicating that the samples with a high amount of gel solution presented high viscosity and density values, with a low gas hold-up percentage, and in addition, the samples were more resistant to mechanical changes during the agitation process. 

Therefore, M5 presents better characteristics than the others aerated CBJ–gelatin gels samples, identifying 8 g of gelatin gel and 40 g of CBJ as ideal ingredients in the final elaboration, since the viscosity and density values were close to the range established for a commercial gel product, with the highest aeration level inside the gel structure (67%).

### 3.2. Texture Profile Analysis (TPA)

The textural characteristics at day 0 and day 28 of aerated CBJ–gelatin gel are shown in Table 3.

Firstly, the samples had a significant decrease in TPA values as the amount of gelatin gel solution decreased and the amount of CBJ increased. Hence, at day 1, M1 had TPA values of approximately 33.4 N, 0.94, 0.91, 30.5 N and 28.5 N, for hardness, springiness, cohesiveness, gumminess and chewiness, respectively, while M5 (the sample with the lowest TPA values) presented values less than 69%, 5%, 17%, 74% and 75% of M1, respectively. Additionally, all the TPA values at day 1 were higher (with statistical differences) than those in samples during 28 days under storage. These maximum values in M1 may be associated with the gelatin gel solution, since a high amount of gel solution causes a more ordered, firm and dense structure, allowing higher resistance to compression force and better recovery to its initial form than the other samples with a low amount of gel solution in the elaboration of aerated CBJ–gelatin gels [52]. Consequently, the downward trend in TPA values from M2 to M5 can be explained by the dilution effect with the increase in the amount of CBJ and the decrease in the amount of gelatin gel solution, and thus the polymer chains progressively lose their rigidity due to the weakening in the connections of the gel molecules for the contrary amounts of gelatin gel solution and CBJ. These results indicate that M5 can be easily chewed, since it had a weaker structure than the other samples. Furthermore, M5 had a soft texture, requiring less energy to swallow, and it had more difficulties in recovering from the first compression [53]. Therefore, the textural properties are influenced by the combination of gelatin gel solution, water content and the amount of each ingredient [54]. 

An important point, the CBJ–gelatin gel solution compromised its structure due to the air incorporation, since the air bubbles occupy different parts in the cross-sectional area, causing empty spaces, and thus the final matrix is an unstable structure against external pressure factors [55]. Furthermore, as the days passed, the samples presented an inferior structural stability and gradual deformity of the typical 3D network due to the high syneresis rate of the cryoconcentrated juice from the gelatin gel structure [56].

A similar tendency was observed by Jakubczyk et al. [7] and Ellis et al. [57], who studied the incorporation of ingredients in aerated gel matrices, clarifying that the highest TPA properties values were obtained on the first days under storage due to strong interactions in the gelling process, and these values decreased as the days progressed due to factors such as temperature, exposure light/oxygen, pH changes and syneresis, among others. 

Therefore, the mixture of gelatin gel, cryoconcentrated juice and 100 g of water allows obtaining a sample with excellent mechanical properties and high stability during refrigerated storage, and thus the combination of ingredients could be selected to produce a novel and interesting aerated gelatin gel enriched with cryoconcentrated juice.

### 3.3. Bubble Size Quantification

**Figure 1:**
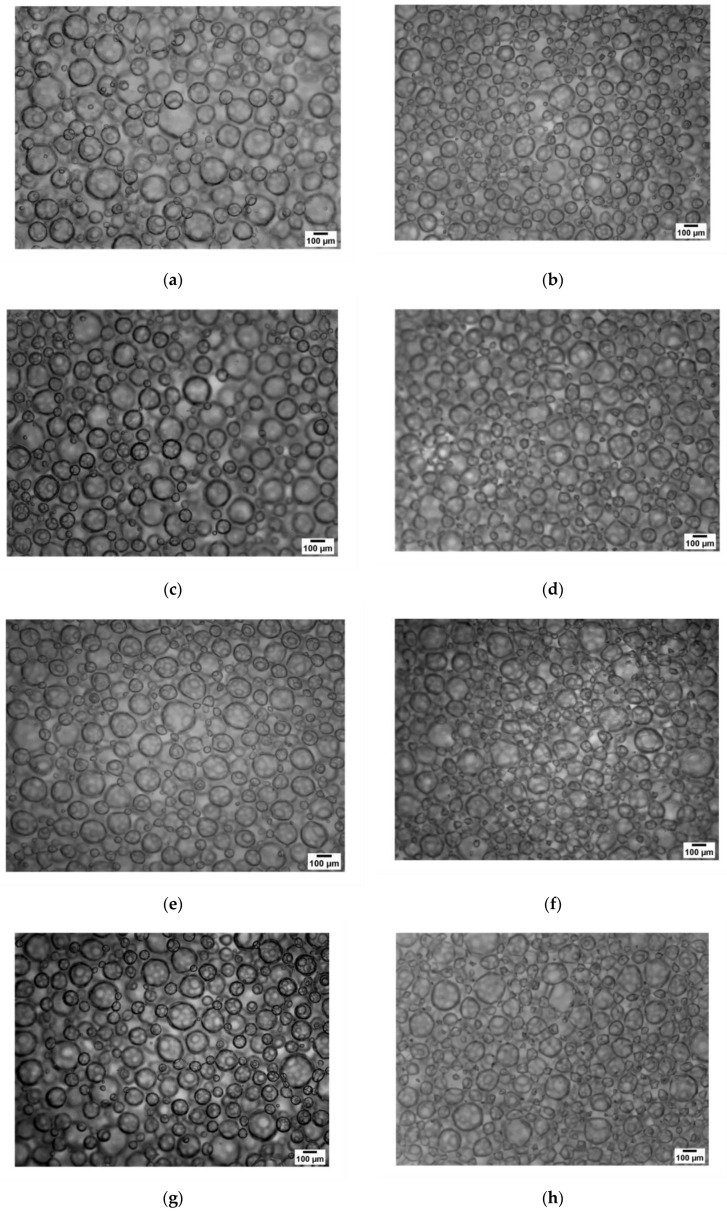

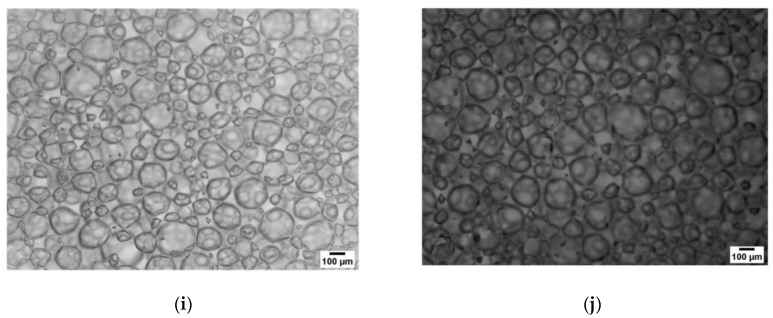
Optical microscopy (100×) of aerated CBJ–gelatin gels under different days of storage: (**a**) M1, day 1; (**b**) M1, day 28; (**c**) M2, day 1; (**d**) M2, day 28; (**e**) M3, day 1; (**f**) M3, day 28; (**g**) M4, day 1; (**h**) M4, day 28; (**i**) M5, day 1; (**j**) M5, day 28.

Figure 1 displays the microstructure of aerated CBJ–gelatin gels at day 1 and day 28. In general, all the samples showed small and large air bubbles. However, the air bubbles presented diverse shapes (spherical and non-spherical) that depended on the gelatin gel/CBJ combination used and the days under storage.

Qualitatively, at day 1, M1 and M2 presented air bubbles with a marked spherical shape. Subsequently, as the amount of gelatin gel solution decreased and the amount of CBJ increased, M3, M4 and M5 had a decrease in the size and a systematic deformation of the air bubbles. Thus, M5 contained numerous small bubbles with a heterogeneous and non-spherical shape distribution. This progressive deformation could be attributed to the decrease in the amount of gel solution from M1 to M5, since a low amount of gel solution produces a loss of the internal network stability, allowing greater formation of air bubbles due to their low resistance of the CBJ–gelatin gel solution in the stirring process. Therefore, the gradual decrease in the amount of gel exerts reduced and non-homogeneous pressure on the air bubbles contained within the sample, explaining the visualization of deformed air bubbles [58].

At day 28, a significant decrease in the sizes and a clear deformation of the air bubbles was observed with respect to the correspondent sample at day 1. These phenomena could be associated with the increase in the syneresis rate of the CBJ and gelatin gel solution from the matrix, i.e., as days passed, there is a continuous drain to the outside of the liquid from the polymeric network, and thus this effect produces the collapse of the structure, generating varied pressure that deforms the air bubbles. Hence, the visual aspect observed in the samples under storage could be linked to the texture profile analysis values, since the TPA values at day 28 were lower than at day 1, indicating a weakening of the structure as the days progressed [58].

Comparable results were pronounced by Ellis et al. [57], who studied the incorporation of different sugars in agar fluid gels and their effect on the microstructure, mentioning that the gel strength was compromised as the days advanced, with a very unstable microstructure at the end of the days under storage.

**Figure 2:**
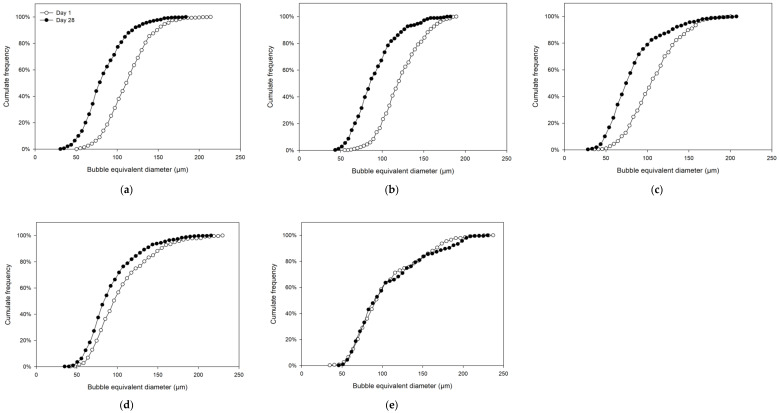
Optical microscopy (100×) of aerated CBJ–gelatin gels under different days of storage: (**a**) M1; (**b**) M2; (**c**) M3; (**d**) M4; (**e**) M5.

Quantitatively, in terms of bubble size distribution (Figure 2), on both days (1 and 28), a decrease in the size of the air bubbles was detected, and in addition, the results allowed corroborating what is shown above in Figure 1, since the air bubbles at day 28 were smaller than the samples at day 1, as more than 50% of the air bubbles at day 28 had an equivalent diameter lower than 74, 79, 83, 85 and 90 µm, while the samples at day 1 presented a low cumulative frequency with the same equivalent diameter as day 28, with 6%, 9%, 13%, 36% and 51%, for M1, M2, M3, M4 and M5 samples, respectively. Thus, M1, M2 and M3 at day 28 had significant differences in the size of air bubbles in relation to day 1 (Figure 2a–c). However, for M4 (Figure 2d), the differences in the air bubbles size were lower than M1, M2 and M3, i.e., M4 had close size distribution values between day 1 and day 28, and finally, M5 did not present differences in the size of the air bubbles between the days (1 and 28).

These values indicated an increase in small bubbles as the amount of gelatin gel solution decreased and the amount of CBJ increased, showing that the minimum amount of gelatin gel (8 g) had lower resistance to the mechanical agitation (air bubbles) in the CBJ–gelatin gel solution than the other samples. Therefore, an increase in the gas hold-up leads to the formation of small bubbles, verifying the relationship indicated by Valenzuela and Aguilera [59], in which the gas hold-up is strongly connected to the bubble size in organic and inorganic samples.

### 3.4. Color Measurement

**Figure 3:**
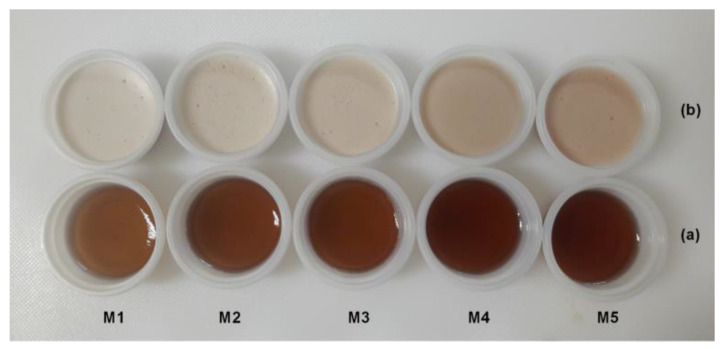
Color changes of gelatin gels: (**a**) CBJ–gelatin gels; (**b**) aerated CBJ–gelatin gels.

Color is a quality parameter used as an indicator of selection and evaluation in different foods, and it has profound influences on consumer acceptability [60]. As shown in Figure 3, a comparison between CBJ–gelatin gels and aerated CBJ–gelatin gels at day 1 was performed to denote the effects of air bubbles inside the CBJ–gelatin gel solution after the gelling process. Initially, as the amount of gel solution decreases and the amount of CBJ solution increases, CBJ–gelatin gels present a light red color (M1) that gradually darkens to an intense red color (M5) due to the high amount of CBJ, and in addition, the cryoconcentrated juice can be recognized by the intense final color obtained via CBCC [61]. Meanwhile, aerated CBJ–gelatin gel exhibits a pale red color with an opaque appearance (M1) that significantly darkens with respect to the gelatin gel/CBJ combination, and thus M5 shows an opaque red color. This difference between aerated and non-aerated gels can be explained by the incorporation of air bubbles, since the air bubbles diffract visible light due to their larger size than the visible wavelength [58]. These results are similar to those mentioned by Zúñiga et al. [55], who reported a color change from yellowish-transparent in non-aerated gels to an opaque white color in aerated gels, concluding that the color change can be due mainly to the addition of air bubbles, since the air bubbles deflect the light.

**Figure 4:**
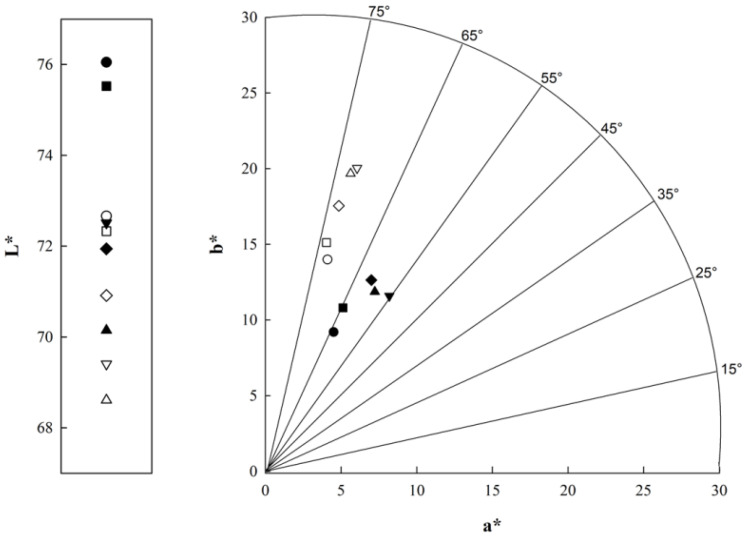
Color changes of aerated CBJ–gelatin gels in the L*, a* and b* plane. M1 (o); M2 (□); M3 (◊); M4 (Δ); and M5 (∇). Sample at day 1 (black color) and storage at day 28 (white color).

Figure 4 displays the color parameters in aerated CBJ–gelatin gel at day 1 and day 28 under the storage period. 

Firstly, significant changes were detected between samples on the same day, indicating that the differences observed were quantitative and qualitative. In general, as the amount of gelatin gel decreased and the amount of CBJ increased, i.e., from M1 to M5, the gels presented a decrease in L*, and an increase in a* and b* values, showing a progressive darkening with a reddish color. Hence, these results corroborate the changes produced by adding a highly colored juice into a transparent or opaque sample (Figure 3).

Specifically, on the days under storage, each sample presented a lower L* value than its corresponding sample at day 1, specifying that the samples acquired darkening in relation to the initial aerated CBJ–gelatin gel (day 1) as the days progressed. In the same way, for a* values, the samples did not show significant differences, i.e., there are no variations between day 1 and day 28 with respect to the green–red axis. Instead, in terms of b* values, an opposite effect was observed, since the samples (M1, M2 and M3) at day 28 had an increase between 25% and 40% in comparison to the samples at day 1, implicating that the samples (day 28) tended to be more yellowish-brown color, and M4 and M5 presented the most significant color changes. This effect could be accredited to the anthocyanin degradation, since anthocyanins can be easily degraded by factors associated with exposure to oxygen (air bubbles), light, temperature and pH changes, causing a change from the natural color to a brown color [62]. 

In terms of ΔE* values, each sample was analyzed to assess color changes between day 1 and day 28. Thus, the values were close to 6.2, 5.6, 5.5, 8.1 and 9.3 CIELab units for M1, M2, M3, M4 and M5, respectively, establishing that the human eye can find differences between the samples based on the scale proposed by Rivero et al. [63] (ΔE* > 3). Comparable results were reported by Guerra-Valle et al. [32] and Jaster et al. [33], who added cryoconcentrated juice to apple slices and natural yogurt, respectively, stipulating that the final food matrix had a similar color to the cryoconcentrated juice. In addition, Hanani et al. [64] and Kia et al. [65] described identical behavior when incorporating phenolic-rich extracts from pomegranate peel and red beet into transparent gelatin gel-based products, respectively.

### 3.5. Determination of the Total Bioactive Compounds Content and Antioxidant Activity

**Figure 5:**
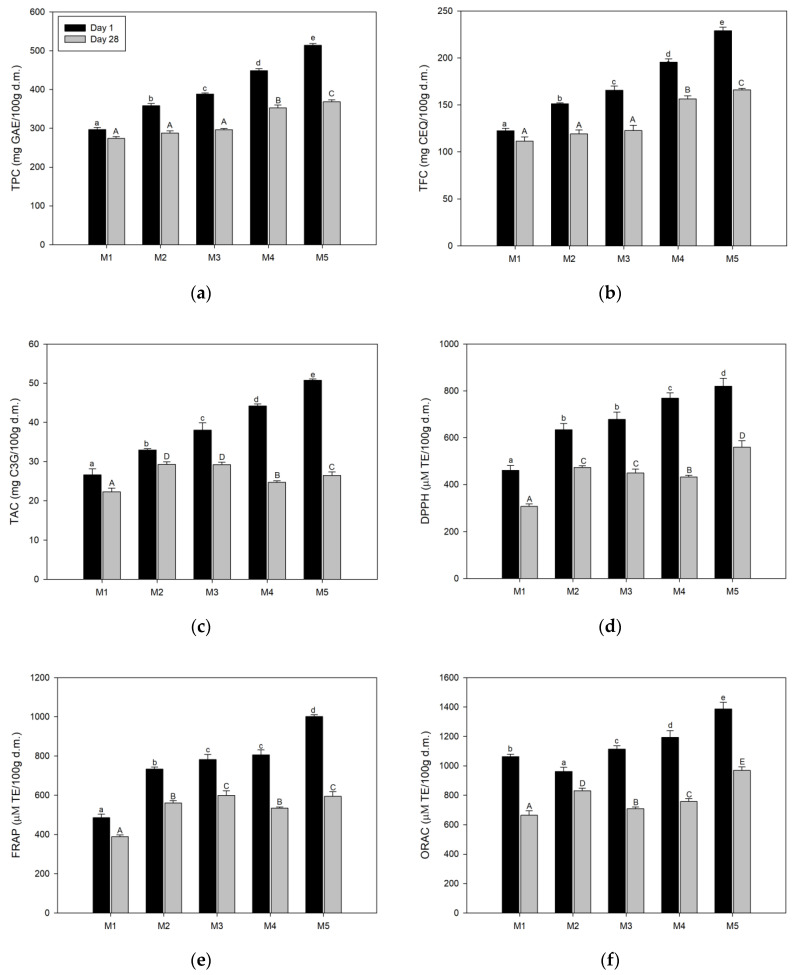
Total bioactive compounds content (TBCC) and antioxidant activity (AA) in aerated CBJ–gelatin gels at day 1 and day 28: (**a**) total polyphenol content (TPC); (**b**) total flavonoid content (TFC); (**c**) total anthocyanin content (TAC); (**d**) 2,2-diphenyl-1-picrylhydrazyl (DPPH); (**e**) ferric reducing antioxidant power (FRAP); (**f**) oxygen radical absorbance capacity (ORAC). a–e: Different small letters denote differences at 5% from M1 to M5 at day 1, according to the LSD test. A–E: Different capital letters denote differences at 5% from M1 to M5 at day 28, according to the LSD test.

Figure 5 shows the TBCC and AA values of the aerated CBJ–gelatin gel at day 1 and day 28. An important point, the fresh blueberry juice had TBCC and AA values of approximately 120 mg GAE/100 g d.m. for TPC, 10 mg C3G/100 g d.m. for TAC and 25 mg CEQ/100 g d.m. for TFC, and 170, 215 and 605 μM TE/100 g d.m. for DPPH, FRAP and ORAC, respectively. The TBCC values were lower than those previously reported by Pertuzatti et al. [66], who studied various bioactive compounds in different blueberry varieties. These differences might be due to the blueberry variety, climatic circumstances, ripening stage, harvest and storage conditions and the methodology applied during the preparation of the fresh juice [67]. Instead, CBJ exhibited values close to 300 mg GAE/100 g d.m., 42 mg C3G/100 g d.m. and 130 mg CEQ/100 g d.m. for TPC, TAC and TFC, and 530, 930 and 2300 µM TE/100 g d.m. for DPPH, FRAP and ORAC, respectively. Therefore, the CBJ results reinforce that the low temperatures used in CBCC allow for increases in various bioactive components [61].

Precisely, at day 1, a significant increase in TBCC (Figure 5a–c) and AA (Figure 5d–f) values was observed as the amount of CBJ increased and the amount of gelatin gel decreased, with values close to 297–514 mg GAE/100 g d.m., 27–51 mg C3G/100 g d.m. and 123–229 mg CEQ/100 g d.m., from M1 to M5, respectively. Hence, the highest values were detected in M5, corresponding to an increase of 173%, 189% and 186% compared to M1, for TPC, TAC and TFC, respectively. For AA, the DPPH and FRAP values were of approximately 461 and 486 µM TE/100 g d.m., 634 and 734 µM TE/100 g d.m., 678 and 782 µM TE/100 g d.m., 768 and 806 µM TE/100 g d.m. and 819 and 1001 µM TE/100 g d.m., for M1, M2, M3, M4 and M5, respectively. Thus, in general, the TBCC and AA values in M5 were over 1.7, 1.4, 1.3 and 1.1, and 1.8, 1.3, 1.2 and 1.1 times higher than M1, M2, M3 and M4, respectively, showing that the high CBJ added in M5 (40 g) provides a considerable amount of bioactive components, and, in turn, demonstrating that the gelatin gel/CBJ combination in M5 resists the methodology applied better than the other samples, since the temperature for the complete incorporation of ingredients inside the gelatin gel solution was ≈40 °C, and the literature mentions that a temperature above 30 °C affects the TBCC and AA stability and produces a decrease in the initial content [68]. In particular, a different trend was observed in the ORAC assay (Figure 5f), since M2 presented a slight decrease with respect to M1. However, from M3, ORAC values had an ascending tendency, which agrees with the DPPH and FRAC trends. Likewise, M5 indicated the highest ORAC value, with approximately 1387 µM TE/100 g d.m., which is equivalent to an increase over 1.3, 1.4, 1.2 and 1.2 times in relation to M1, M2, M3 and M4, respectively.

Different studies have reported that the addition of cryoconcentrated juice helps to enrich various food matrices with low bioactive components such as apple slices [32] and natural yogurt [33], increasing up to two and three times the initial bioactive compound contents, respectively. Therefore, these results indicate that the addition of bioactive components from CBJ can reinforce the initial nutritional value of the matrix sample.

On the days under storage, a general reduction in the TBCC and AA values was perceived in comparison to the samples at day 1. From M1 to M5, the samples had an ascending performance in TPC and TFC, with values less than 8%, 20%, 24%, 21% and 28%, and 16%, 11%, 23%, 44% and 48% of M1, M2, M3, M4 and M5 at day 1, respectively. However, in TAC, M3, M4 and M5 had a significant decay, implicating a pronounced degradation close to 1%, 16% and 10% in relation to M2, respectively. In the same way, an irregular comportment was observed in AA terms, since M3 and M4 in DPPH, M4 and M5 in FRAP and M3 and M4 in ORAC presented a decrease in comparison to M2, M3 and M2, respectively. The degradation and unstable behaviors of TBCC and AA can be accredited to the influences of numerous factors such as temperature, light, humidity, initial concentration, pH and oxygen, among others [62]. Thus, the air bubbles inside gelatin gel samples accelerate the TBCC and AA degradation, since the oxygen reacts with antioxidant substances such as anthocyanin through hydrogen atom donation of the hydroxyl group to a free radical, leading to a decrease in the anthocyanin content [69]. These lower results at day 28 than day 1 indicate oxidation and polymerization of the bioactive components as days passed, and, in turn, this can be connected to the distinctive loss of antioxidant activity [61].

In terms of RP (Table S1), the TBCC retention at day 1 was higher than the samples at day 28. Specifically, the samples presented, as a minimum, an RP value close to 84%, 48% and 83% for TPC, TAC and TFC, respectively. The high RP values at day 1 can be related to the immediate incorporation of the cryoconcentrated juice into the gelatin gel solution. Later, as the days advance, a continuous TBCC degradation occurs in the samples due to the various factors described above, and it corroborates the decrease in RP values at day 28.

To date, there are no studies about the interaction of a cryoconcentrated juice and gelation gel solution. However, our RP results are in agreement with those reported by Orellana-Palma et al. [14] and Correa et al. [70] in apple juice and aqueous coffee extract, with RP values close to 90% and 85%, respectively. Hence, the RP values showed the positive effects of the subzero concentration technique to preserve susceptible and thermolabile bioactive compounds from the fresh juice, and thus these samples can be added in a solid matrix to enrich the initial bioactive compound contents.

### 3.6. Analysis of Individual Phenolic Compounds (IPC) by HPLC

Blueberries have been widely recognized as an important source of phenolic compounds such as anthocyanins, flavonoids and phenolic acids, among others [22,23]. In our case, phenolic acids (44%) were the most abundant phenolic compounds in the blueberry juice, followed by anthocyanins (32%) and flavonoids (24%). Specifically, the three predominant IPC in blueberry juice were caffeic acid (59 mg/100 g d.m.), maldivin (20 mg/100 g d.m.) and delphinidin (16 mg/100 g d.m.). Thus, the values are in accordance with Sellappan et al. [71], who noted that the principal IPC in blueberries were malvidin (47%), delphinidin (37%) and cyanidin (16%). In other study, Zhou et al. [72] reported that malvidin, petunidin and delphinidin are the main IPC in the same fruit. Therefore, these different results could be due to modifications in the geographical place of growth, weather conditions, fruit maturity, type of harvest and pre- and post-harvest processing, and, in addition, the variations in IPC values could be associated with the changes in the environmental and seasonal conditions each year [73]. In terms of CBJ, all the IPC values significantly increased at each cycle. Thus, in the last cycle, the values were over 1.8, 3.5 and 4.0 times higher than the fresh juice, for flavonoids, phenolic acids and anthocyanins, respectively. This behavior is consistent with the increase in CBJ on TPC, TFC and TAC previously described (Section 3.5). In the same way, Adorno et al. [18] also observed an increase up to 4.0 and 6.0 times in various bioactive components in the CC applied to strawberry juice. Furthermore, studies on CC applied to fruit juices have reported a significant increase in monomers [22], anthocyanins [34], polyphenols [38], flavonoids [61], antioxidant capacity [14] and volatile compounds [74].

The IPC values in each aerated gelatin gel/CBJ combination are shown in Table 4. Firstly, all the samples at day 1 had significant differences in relation to day 28. Thus, as days passed, the samples at day 28 had a loss between 2.3% and 22.8%, 48.7% and 68.1%, 3.7% and 15.6%, 3.4% and 28.0%, 68.6% and 85%, 1.5% and 32.6%, 1.0% and 9.6% and 0.4% and 7.6%, for delphinidin, cyanidin, malvidin, epigallocatechin gallate, epicatechin, quercetin, myricetin and caffeic acid, in comparison to the IPC values at day 1, respectively. These low IPC values at day 28 could be linked to the loss of 3D network stability, resulting in a progressive weakening of the collagen molecules that generates the gel network, thus increasing the high loss of bioactive components (syneresis) from the aerated matrix structure as the days under storage advance. The results are in agreement with those reported by Ali et al. [75], who studied the influence of gelatin bags on characteristics of fried fish stored for 30 days, explaining that the loss of various components through the gelatin films can be connected to the weakening of the gelatin structure as the days passed.

Moreover, at day 1 and day 28, the IPC values presented an irregular behavior from M1 to M4. However, for anthocyanins and phenolic acids, M5 had the highest IPC values, corroborating that the highest amount of CBJ (40 g) allows higher bioactive compounds retention than the other gelatin gel/CBJ combinations. Instead, for flavonoids, from M1 to M5, an irregular performance was observed in all the samples, indicating that the flavonoids inside the aerated CBJ–gelatin gel had different interactions in the preparation steps, gelling process and storage conditions, and thus, depending on the external conditions, these bioactive compounds can increase or decrease in the final sample.

An important point, there are limits in the incorporation of ingredients inside aerated gelatin gel, since Zhao and Sun [76] mentioned that a high polyphenols concentration inside a gelatin gel matrix provokes the precipitation of collagen molecules and, in turn, produces the collapse of the gelatin gel network, as well as decreasing the bioactive compounds content (syneresis), explaining the high loss in IPC values as days passed.

As can be observed from Table S2, at day 1, the RP values had slight differences from M1 to M5. Thus, flavonoids had the highest RP values (48% to 93%), while anthocyanins and phenolic acids presented values between 3% and 68% and 13% and 19%, respectively. These differences may be related to the molecular structure in each IPC. Specifically, each IPC has various phenolic groups, and thus these molecules have high antioxidant capacity. However, each group has been recognized due to their reaction to oxidation and/or changes during the storage time [69], explaining the low RP values in some specific compounds. After 28 days of storage, the samples exhibited lower RP values than samples at day 1. These values could be connected to the time exposition under the refrigeration temperature. Therefore, these results demonstrate that aerated CBJ–gelatin gels are valuable sources of individual phenolic compounds, improving the nutritional value of the gelatin gel.

## 4. Conclusions

The combination of 8 g gelatin gel and 40 g CBJ (called M5) showed the best values with respect to the other gelatin gel/CBJ combinations. Specifically, M5 had a soft texture, with small and non-spherical air bubbles, and it showed similar behavior to the commercial gel products, in terms of apparent viscosity, density and TPA values, allowing a commercial approach compared to the other samples. Moreover, the samples had an attractive color since the natural fresh blueberry juice color was maintained in the final sample. Furthermore, M5 retained a considerable amount of TBCC, individual phenolic compounds and AA in comparison to the other samples (from M1 to M4) due to the high amount of CBJ used in the elaboration process. Therefore, CBJ can be an interesting functional ingredient to produce a novel gel matrix enriched with concentrated juice at low temperatures; thus, M5 can be an innovative aerated gelatin-based product due to its structural attributes with high nutritional values, and in addition, the sample can be characterized as a food matrix without artificial sweeteners. Finally, the ingredients used in M5 could be used as a basis for future studies in the development of aerated products enriched with cryoconcentrated juice. Additionally, the present study has shown an interesting interaction between a gelatin gel solution and a highly concentrated sample, and thus we suggest future applications for 3D printing, since the CBJ–gelatin gel mix (rich in bioactive components and antioxidant capacity) could be used to coat various matrices. Hence, these 3D-printed products coated with CBJ–gelatin gel could have various potential engineering applications (level-up medical devices or food design), since these mixes have good characteristics of adhesion, biocompatibility and mechanical strength, among others.

## Figures and Tables

**Table 1:** Experimental conditions for the aerated concentrated blueberry juice (CBJ)–gelatin gel elaboration.

Sample	Ingredients (g)		
	Gelatin Gel	CBJ	Water
M1	14	20	100
M2	13	25	100
M3	11	30	100
M4	10	35	100
M5	8	40	100

**Table 2:** Viscosity, density (*ρ*) and gas hold-up (*ε*) of the aerated CBJ–gelatin gels.

	M1	M2	M3	M4	M5
Viscosity (kPa·s)	31.0 ± 1.8 ^e^	19.0 ± 1.1 ^d^	14.22 ± 1.2 ^c^	8.4 ± 0.8 ^ab^	7.1 ± 0.5 ^a^
*ρ_acg_* (kg/m^3^)	511.3 ± 24.3 ^de^	490.5 ± 19.0 ^cd^	456.4 ± 17.9 ^c^	387.6 ± 29.9 ^ab^	349.0 ± 19.6 ^a^
*ε* (%)	49.1 ± 1.1 ^a^	52.1 ± 1.7 ^b^	57.1 ± 1.6 ^c^	64.2 ± 2.0 ^d^	67.1 ± 0.8 ^e^

Different letters in the same row show significant differences at 5% between homogeneous groups in each variable according to a least significant difference test (LSD). *ρ_acg_* is the density of aerated CBJ–gelatin gel.

**Table 3:** Texture profile analysis (TPA) results of aerated CBJ–gelatin gels samples.

	Day	M1	M2	M3	M4	M5
Hardness (N)	1	33.44 ± 0.42 ^e,B^	25.62 ± 0.88 ^d,B^	20.08 ± 0.60 ^c,B^	13.63 ± 0.11 ^b,B^	10.51 ± 0.23 ^a,B^
	7	32.25 ± 1.18 ^e^	24.50 ± 2.06 ^d^	19.44 ± 2.06 ^c^	13.05 ± 0.03 ^b^	10.09 ± 1.04 ^a^
	14	30.59 ± 0.48 ^e^	23.37 ± 0.97 ^d^	17.89 ± 1.22 ^c^	12.26 ± 0.23 ^b^	9.73 ± 0.19 ^a^
	21	28.16 ± 0.60 ^e^	22.56 ± 0.71 ^d^	16.25 ± 0.50 ^c^	11.43 ± 1.25 ^b^	8.33 ± 0.55 ^a^
	28	22.83 ± 1.57 ^e,A^	17.75 ± 1.31 ^d,A^	14.58 ± 0.44 ^c,A^	10.65 ± 0.12 ^b,A^	7.26 ± 0.13 ^a,A^
Springiness	1	0.94 ± 0.00 ^d,B^	0.92 ± 0.01 ^b,c,B^	0.90 ± 0.01 ^a,b,B^	0.89 ± 0.02 ^a,b,B^	0.89 ± 0.01 ^a,B^
	7	0.92 ± 0.04 ^c,d^	0.91 ± 0.01 ^b,c^	0.89 ± 0.02 ^a,b^	0.88 ± 0.02 ^a^	0.88 ± 0.02 ^a^
	14	0.90 ± 0.03 ^c,d^	0.90 ± 0.03 ^c,d^	0.88 ± 0.02 ^c^	0.84 ± 0.01 ^a,b^	0.82 ± 0.02 ^a^
	21	0.88 ± 0.02 ^d,e^	0.87 ± 0.02 ^d^	0.84 ± 0.00 ^c^	0.82 ± 0.00 ^b^	0.80 ± 0.01 ^a^
	28	0.88 ± 0.01 ^d,e,A^	0.86 ± 0.01 ^d,A^	0.82 ± 0.01 ^b,c,A^	0.81 ± 0.01 ^a,b,A^	0.78 ± 0.02 ^a,A^
Cohesiveness	1	0.91 ± 0.02 ^d,e,B^	0.88 ± 0.04 ^c,d,B^	0.87 ± 0.02 ^c,B^	0.80 ± 0.03 ^a,b,B^	0.76 ± 0.02 ^a,B^
	7	0.87 ± 0.02 ^d,e^	0.84 ± 0.02 ^c,d^	0.82 ± 0.03 ^c^	0.75 ± 0.03 ^a,b^	0.73 ± 0.03 ^a^
	14	0.84 ± 0.03 ^d,e^	0.83 ± 0.02 ^c,d^	0.80 ± 0.01 ^c^	0.74 ± 0.01 ^b^	0.71 ± 0.01 ^a^
	21	0.83 ± 0.03 ^d,e^	0.79 ± 0.03 ^c,d^	0.78 ± 0.02 ^c^	0.73 ± 0.02 ^a,b^	0.70 ± 0.02 ^a^
	28	0.81 ± 0.02 ^d,A^	0.76 ± 0.02 ^b,c,A^	0.74 ± 0.01 ^b,A^	0.73 ± 0.03 ^a,b,A^	0.70 ± 0.02 ^a,A^
Gumminess (N)	1	30.53 ± 0.49 ^e,B^	22.60 ± 0.59 ^d,B^	17.46 ± 0.30 ^c,B^	10.84 ± 0.49 ^b,B^	7.98 ± 0.42 ^a,B^
	7	28.15 ± 0.60 ^e^	19.74 ± 1.23 ^d^	16.05 ± 2.09 ^c^	9.56 ± 0.37 ^b^	7.42 ± 1.09 ^a^
	14	25.64 ± 1.02 ^e^	19.03 ± 1.21 ^d^	14.61 ± 0.82 ^c^	8.85 ± 0.13 ^b^	6.68 ± 0.20 ^a^
	21	23.31 ± 0.67 ^e^	17.31 ± 0.17 ^d^	12.60 ± 0.08 ^c^	8.45 ± 0.94 ^b^	5.83 ± 0.38 ^a^
	28	18.44 ± 1.34 ^e,A^	13.49 ± 1.08 ^d,A^	11.48 ± 0.29 ^c,A^	7.82 ± 0.36 ^b,A^	5.06 ± 0.22 ^a,A^
Chewiness (N)	1	28.51 ± 0.51 ^e,B^	20.49 ± 1.04 ^d,B^	16.15 ± 0.35 ^c,B^	9.66 ± 0.78 ^b,B^	7.12 ± 0.23 ^a,B^
	7	25.44 ± 1.20 ^e^	17.87 ± 1.18 ^d^	14.32 ± 2.80 ^c^	8.39 ± 0.27 ^b^	6.51 ± 0.84 ^a^
	14	22.45 ± 1.25 ^e^	17.17 ± 1.53 ^d^	12.79 ± 0.42 ^c^	7.42 ± 0.19 ^b^	5.74 ± 0.27 ^a^
	21	21.29 ± 0.68 ^e^	14.61 ± 0.77 ^d^	10.56 ± 0.05 ^c^	7.12 ± 0.78 ^b^	5.27 ± 0.40 ^a^
	28	16.21 ± 1.25 ^e,A^	12.08 ± 1.09 ^d,A^	9.46 ± 0.27 ^c,A^	7.06 ± 0.39 ^b,A^	4.35 ± 0.36 ^a,A^

^a–e^: Different small letters in the superscript denote differences at 5% between the samples (M1 to M5) for the same phenolic compound at each week, according to the LSD test. ^A,B^: Different capital letters in the superscript denote differences at 5% for the same sample (M1 to M5) and same phenolic compound between day 1 and day 28, according to the Student’s *t*-test.

**Table 4:** Individual phenolic compounds (IPC) of aerated CBJ–gelatin gels at day 1 and day 28 under storage.

	Day	M1	M2	M3	M4	M5
*Anthocyanins*						
Delphinidin	1	3.12 ± 0.04 ^b,c,B^	3.06 ± 0.08 ^a,b,B^	3.02 ± 0.04 ^a,B^	3.35 ± 0.06 ^d,B^	4.12 ± 0.01 ^e,B^
	28	2.97 ± 0.03 ^A^	2.98 ± 0.02 ^A^	2.95 ± 0.02 ^A^	2.94 ± 0.01 ^a,A^	3.18 ± 0.05 ^A^
Cyanidin	1	1.77 ± 0.01 ^a,B^	1.98 ± 0.05 ^b,B^	2.07 ± 0.03 ^c,B^	2.36 ± 0.01 ^d,B^	2.94 ± 0.08 ^e,B^
	28	0.77 ± 0.00 ^c,A^	0.70 ± 0.01 ^b,A^	0.66 ± 0.01 ^a,A^	1.21 ± 0.03 ^d,A^	1.34 ± 0.00 ^e,A^
Malvidin	1	4.88 ± 0.04 ^c,B^	4.58 ± 0.12 ^a,B^	4.63 ± 0.19 ^a,b,B^	4.86 ± 0.26 ^b,c,B^	5.45 ± 0.30 ^d,B^
	28	4.59 ± 0.10 ^d,A^	4.41 ± 0.00 ^c,A^	4.28 ± 0.04 ^a,A^	4.40 ± 0.00 ^b,A^	4.60 ± 0.17 ^d,e,A^
*Flavonoids*						
Epigallocatechin gallate	1	8.17 ± 0.21 ^a,B^	11.01 ± 0.56 ^c,B^	13.64 ± 0.49 ^e,B^	12.24 ± 0.15 ^d,B^	9.18 ± 0.21 ^b,B^
	28	7.89 ± 0.03 ^b,A^	7.93 ± 0.17 ^b,c,A^	10.77 ± 0.11 ^e,A^	9.87 ± 0.28 ^d,A^	7.68 ± 0.17 ^a,A^
Epicatechin	1	3.34 ± 0.00 ^a,B^	3.84 ± 0.17 ^b,A^	4.44 ± 0.13 ^c,A^	5.59 ± 0.07 ^d,A^	6.61 ± 0.10 ^e,A^
	28	1.05 ± 0.03 ^c,A^	0.83 ± 0.01 ^a,A^	1.14 ± 0.01 ^d,A^	0.84 ± 0.02 ^a,b,A^	1.24 ± 0.04 ^e,A^
Quercetin	1	3.99 ± 0.11 ^a,b,A,B^	5.70 ± 0.00 ^e,A^	4.67 ± 0.12 ^d,B^	3.85 ± 0.09 ^a,B^	4.07 ± 0.09 ^b,c,B^
	28	3.93 ± 0.01 ^e,A^	3.84 ± 0.00 ^d,A^	3.81 ± 0.02 ^c,A^	3.67 ± 0.07 ^b,A^	3.58 ± 0.00 ^a,A^
Myricetin	1	2.96 ± 0.03 ^b,c,A,B^	3.10 ± 0.00 ^e,B^	2.81 ± 0.11 ^a,B^	3.05 ± 0.03 ^d,B^	2.92 ± 0.03 ^a,b,B^
	28	2.93 ± 0.06 ^c,A^	3.01 ± 0.04 ^d,e,A^	2.63 ± 0.04 ^a,A^	3.00 ± 0.04 ^c,d,A^	2.64 ± 0.04 ^a,b,A^
*Phenolic acids*						
Caffeic acid (CA)	1	5.07 ± 0.18 ^a,A,B^	5.79 ± 0.07 ^b,B^	5.89 ± 0.02 ^c,B^	5.92 ± 0.07 ^b,c,d,B^	6.11 ± 0.04 ^e,B^
	28	5.05 ± 0.02 ^a,A^	5.35 ± 0.13 ^b,A^	5.61 ± 0.04 ^c,A^	5.65 ± 0.03 ^c,d,A^	5.98 ± 0.01 ^e,A^

^a–e^: Different small letters in the superscript denote differences at 5% between the samples (M1 to M5) for the same phenolic compound, according to the LSD test. ^A,B^: Different capital letters in the superscript denote differences at 5% for the same sample (M1 to M5) and the same phenolic compound between day 1 and day 28, according to the Student’s *t*-test.

## Supplementary Materials

The following are available online at https://www.mdpi.com/2073-4360/12/12/2769/s1, Table S1: RP values of TBCC in aerated CBJ–gelatin gels, Table S2: RP values of IPC in aerated CBJ–gelatin gels.
