# Supplementary material for: Comparative Study of the Structural Properties, Color, Bioactive Compounds Content and Antioxidant Capacity of Aerated Gelatin Gels Enriched with Cryoconcentrated Blueberry Juice during Storage
Source: Polymers (Basel). 2020 Nov 24;12(12):2769. doi: 10.3390/polym12122769 (PMC7760114; doi:10.3390/polym12122769)
Supplement: Supplementary file 1 [file polymers-12-02769-s001.zip › polymers-1009860-supplementary.docx]

**SUPPLEMENTARY MATERIALS**

**Table S1.** RP values of TBCC in aerated CBJ-gelatin gels.

| TBCC | Day | M1 | M2 | M3 | M4 | M5 |
| --- | --- | --- | --- | --- | --- | --- |
| TPC | 1 | 89.5 | 90.0 | 83.6 | 84.5 | 86.0 |
|  | 28 | 82.6 | 72.2 | 63.8 | 66.4 | 61.7 |
| TAC | 1 | 48.4 | 49.5 | 49.0 | 50.3 | 50.4 |
|  | 28 | 40.5 | 44.0 | 37.6 | 28.1 | 26.3 |
| TFC | 1 | 86.0 | 88.5 | 83.1 | 85.8 | 89.3 |
|  | 28 | 78.2 | 69.7 | 61.5 | 68.6 | 64.7 |

**Table S2.** RP values of IPC in aerated CBJ-gelatin gels.

|  | **Day** | **M1** | **M2** | **M3** | **M4** | **M5** |
| --- | --- | --- | --- | --- | --- | --- |
| *Anthocyanins* |  |  |  |  |  |  |
| Delphinidin | 1 | 67.7 | 63.6 | 53.8 | 64.2 | 66.4 |
|  | 28 | 64.1 | 61.9 | 52.5 | 45.9 | 44.1 |
| Cyanidin | 1 | 11.8 | 11.0 | 9.9 | 9.8 | 10.9 |
|  | 28 | 5.1 | 3.9 | 3.2 | 5.1 | 5.0 |
| Malvidin | 1 | 48.6 | 46.9 | 46.3 | 43.4 | 43.3 |
|  | 28 | 42.1 | 44.0 | 36.0 | 30.2 | 28.1 |
| *Flavonoids* |  |  |  |  |  |  |
| Epigallocatechin gallate | 1 | 82.9 | 93.1 | 93.8 | 87.6 | 84.8 |
|  | 28 | 80.0 | 77.0 | 78.1 | 62.5 | 53.3 |
| Epicatechin | 1 | 51.1 | 48.8 | 48.4 | 53.3 | 56.1 |
|  | 28 | 16.0 | 10.6 | 14.6 | 8.0 | 10.6 |
| Quercetin | 1 | 92.7 | 90.3 | 87.4 | 85.9 | 82.5 |
|  | 28 | 91.2 | 74.3 | 63.2 | 57.6 | 50.0 |
| Myricetin | 1 | 70.9 | 61.9 | 68.1 | 65.7 | 68.9 |
|  | 28 | 62.6 | 54.0 | 50.2 | 45.0 | 35.1 |
| *Phenolic acids* |  |  |  |  |  |  |
| Caffeic acid (CA) | 1 | 16.4 | 15.5 | 15.5 | 17.8 | 18.9 |
|  | 28 | 14.0 | 13.5 | 13.1 | 14.5 | 14.1 |
